# Regulation of JAM2 Expression in the Lungs of Streptozotocin-Induced Diabetic Mice and Human Pluripotent Stem Cell-Derived Alveolar Organoids

**DOI:** 10.3390/biomedicines8090346

**Published:** 2020-09-11

**Authors:** Roya Rasaei, Eunbi Kim, Ji-Young Kim, Sunghun Na, Jung-Hyun Kim, Jinbeom Heo, Dong-Myung Shin, Sun Shim Choi, Seok-Ho Hong

**Affiliations:** 1 Department of Internal Medicine, School of Medicine, Kangwon National University, Chuncheon 24341, Korea; royarasaei93@gmail.com (R.R.); myeunbi90@naver.com (E.K.); jeans330910@gmail.com (J.-Y.K.); katop2024@naver.com (J.-H.K.); 2 Department of Obstetrics and Gynecology, School of Medicine, Kangwon National University, Chuncheon 24341, Korea; lahun@kangwon.ac.kr; 3 Department of Biomedical Sciences, Asan Medical Center, University of Ulsan College of Medicine, Seoul 05505, Korea; hjb0328@naver.com (J.H.); d0shin03@amc.seoul.kr (D.-M.S.); 4 Division of Biomedical Convergence, College of Biomedical Science, Institute of Bioscience & Biotechnology, Kangwon National University, Chuncheon 24341, Korea; schoi@kangwon.ac.kr

**Keywords:** hyperglycemia, JAM2, lung, hPSC, alveolar organoid

## Abstract

Hyperglycemia is a causative factor in the pathogenesis of respiratory diseases, known to induce fibrosis and inflammation in the lung. However, little attention has been paid to genes related to hyperglycemic-induced lung alterations and stem cell applications for therapeutic use. In this study, our microarray data revealed significantly increased levels of junctional adhesion molecule 2 (JAM2) in the high glucose (HG)-induced transcriptional profile in human perivascular cells (hPVCs). The elevated level of JAM2 in HG-treated hPVCs was transcriptionally and epigenetically reversible when HG treatment was removed. We further investigated the expression of JAM2 using in vivo and in vitro hyperglycemic models. Our results showed significant upregulation of JAM2 in the lungs of streptozotocin (STZ)-induced diabetic mice, which was greatly suppressed by the administration of conditioned medium obtained from human mesenchymal stem cell cultures. Furthermore, JAM2 was found to be significantly upregulated in human pluripotent stem cell-derived multicellular alveolar organoids by exposure to HG. Our results suggest that JAM2 may play an important role in STZ-induced lung alterations and could be a potential indicator for predicting the therapeutic effects of stem cells and drugs in diabetic lung complications.

## 1. Introduction

A chronic hyperglycemic condition has widespread adverse effects on various tissues, including the heart, kidney, retina, muscle, liver, and vasculature [1]. Hyperglycemia triggers the infiltration of immune cells in multiple organ tissues and the production of reactive oxygen species, which result in an increased proinflammatory process and oxidative stress [2,3]. The persistence of inflammatory states and oxidative stress lead to the inflexibility of vessels and vascular leakage and induce fibrotic changes by promoting epithelial to mesenchymal transition (EMT) in multiple tissues [4] The vascular dysfunction and EMT process play a central role in developing diabetic complications, suggesting that the functions and structure of the lung are also affected by diabetes mellitus (DM) because the alveolar–capillary system is characterized by a great microvascular and epithelial reserve. However, little attention has been paid to pulmonary pathologies in the context of DM.

Recent studies on streptozotocin (STZ)-induced diabetic animal models reported that DM induces fibrotic changes in the lung, which is mediated through TGF (Transforming growth factor)-β-activated EMT signaling pathways [5]. The antioxidant activity in the lungs of diabetic rats was significantly reduced, suggesting that STZ-induced oxidative stress plays a key role in the pathogenesis of diabetic lung tissue. In fact, the treatment of antioxidants such as N-acetylcystein and Expendin-4 in diabetic animals was found to contribute to the attenuation of STZ-induced lung damage [6,7]. In humans, the pulmonary function test suggested a reduction in the lung functions, including total lung capacity, forced vital capacity, diffusing capacity and respiratory muscle strength in type 1 and/or 2 DM patients [8]. These results link the functional abnormalities in the respiratory tract of DM patients with the development of diverse pulmonary diseases. Meta-analyses, retrospective and cohort studies have shown that DM patients have a higher risk of developing pulmonary diseases including lung cancer, tuberculosis, asthma, pulmonary fibrosis (PF) and chronic obstructive pulmonary disease (COPD) [9,10]. While all these findings indicate a positive correlation between DM and lung disorders, the underlying pathophysiological mechanisms for lung dysfunction in patients with DM and therapeutic strategies are not completely understood due to the lack of accessibility of human diabetic lung tissues and potential indicators of dysfunction in diabetic lung tissue.

In the present study, we defined a set of genes with hyperglycemia-upregulated expression in human perivascular cells (hPVCs), as the primary target of the hyperglycemia-induced adverse effects, using DNA microarrays. Among the genes, junctional adhesion molecule 2 (*JAM2*) was found to be transcriptionally and epigenetically regulated by hyperglycemia. We further investigated the regulation of JAM2 expression using in vivo and in vitro hyperglycemic models and evaluated it as a potential indicator for predicting diabetic lung complications and therapeutic effects of multipotent stem cells.

## 2. Materials and Methods

### 2.1. hPVC Cultures and HG Treatment

PVCs were isolated from human umbilical cords and cultured as previously described [11]. hPVCs were obtained from full-term deliveries by cesarean section with donors’ written consent (Institutional Review Board No. 2014-06-003-010; Kangwon National University Hospital). hPVCs were treated with normal glucose (NG, 5 mM) and high glucose (HG) (25 and 50 mM) for the indicated periods.

### 2.2. Cell Proliferation Assay

The short- and long-term growth properties of hPVCs under hyperglycemic conditions were analyzed as previously described [12].

### 2.3. hPVC Differentiation

The multilineage differentiation potential of hPVCs cultured in NG and HG conditions was evaluated at passages 3 and 4 as previously described [13]. Briefly, for adipogenic differentiation, hPVCs (4 × 10^4^ cells/well) were plated in 12-well tissue culture plates and cultured in adipogensis differentiation medium (A10070-01; StemPro^®^, Thermo Fisher Scientific, Waltham, MA, USA) for 21 days. After 21 days, the cultures were stained with Oil Red O (CM-005; LifeLine Cell Technology, CA, USA) to visualize the intracellular accumulation of lipid vacuoles. For osteogenic differentiation, hPVCs (4 × 10^4^ cells/well) were seeded in 12-well tissue culture plates and cultured in osteogenesis differentiation medium (A100-01; StemPro^®^, Thermo Fisher Scientific, Waltham, MA, USA) for 21 days. On day 21, the cells were stained with Alizarin Red S (Lifeline Cell Technology) to visualize the mineralization in osteogenic cell cultures.

### 2.4. Preparation of MSC-CM

Conditioned medium (CM) from human mesenchymal stem cells (MSCs) was prepared as previously described [14]. Briefly, MSCs were plated on 100-mm dishes and cultured until they reached 80–90% confluence. The cells were incubated with serum-free α-MEM for 24 h at 37 °C in a humidified atmosphere with 5% CO_2_. The conditioned medium was harvested and passed through a 0.22-μm filter. The filtered medium was concentrated using an Amicon Ultra-15 Centrifugal Filter Units (3-kDa cutoff membrane, Millipore, Burlington, MA, USA) and stored at −80 °C until use.

### 2.5. Mice and CM Administration

Male C57BL/6J mice were purchased from Dooyeol Biotech (Seoul, Korea) and housed in a specific pathogen-free facility. All animal studies were approved by the Institutional Animal Care and Use Commitment of Kangwon National University (KW-180809-2, approved on 9 August 2018). Mice (18–20 g, 8 weeks) were intraperitoneally injected with 50 mg/kg STZ (S0130, Sigma-Aldrich, Burlington, MA, USA) daily for 5 consecutive days to induce type 1 DM [2]. After 5 days, glucose levels were evaluated using Accu-CHECK Active (Roche Diagnostic Inc., Korea). Mice with a basal blood glucose level of more than 250 mg/dL were considered diabetic and included in experiments. After induction of diabetes, mice were intravenously administered with MSC-CM (40 μg/100 μL) for 6 weeks, whereas control animals received intravenous saline.

### 2.6. Morphological Analysis of the Mouse Lung

Mouse lung tissue was stained with hematoxylin and eosin (H&E) to analyze the histological structure following the induction of hyperglycemia. Eight random fields were taken to evaluate alveolar wall thickness and alveolar size using digital imaging software (NIH Image J) as previously described [15].

### 2.7. Immunofluorescence Staining

For immunofluorescence staining, 4-μm-thick lung sections were dewaxed with xylene and rehydrated with a gradient of ethanol. The sections were subjected to antigen retrieval with a citrate buffer bath (pH 6) at boiling temperature and then blocked for endogenous peroxidase activity. After washing with phosphate-buffered saline (PBS), the sections were incubated with primary antibodies against alpha smooth muscle actin (α-SMA) (SC-53015; Santa Cruz Biotechnology, Dallas, TX, USA), JAM2 (ab139645; Abcam, Cambridge, UK), Neutrophil (Neu) (ab2557; Abcam, Cambridge, UK), Macrophage (Mac) (ab56297; Abcam, Cambridge, UK), and dendritic cell (DC) (NB110-85474; Novus Biologicals, Toronto, ON, Canada) overnight at 4 °C. The sections were rinsed with PBS and incubated with secondary FITC- or RFP-labelled antibodies for 30 min. Nuclei were counterstained with 4′,6-diamidino-2-phenylidone (DAPI) and the fluorescence images were captured with a fluorescence microscope (IX-51, Olympus, Japan).

### 2.8. Microarray and Data Analysis

Total RNA was extracted from hPVCs using the RNeasy Mini Kit (74106; Qiagen, MD, USA). RNA purity and integrity were evaluated using the Agilent 2100 Bioanalyzer (Agilent Technologies, Palo Alto, CA, USA). The Affymetrix Whole transcript expression array process was executed according to the manufacturer’s protocol (GeneChip Whole Transcript PLUS Reagent Kit, Thermo Fisher Scientific, Waltham, MA, USA). The data were summarized and normalized using the robust multi-average (RMA) method implemented in the Affymetrix Power Tools. The results produced by gene-level RMA analysis were exported and subjected to differentially expressed gene (DEG) analysis. The statistical significance of the expression data was determined using an independent Student *t*-test and fold change methods in which the null hypothesis was defined as no observed difference between the groups. A hierarchical cluster analysis of the DEGs was performed using complete linkage and Euclidean distance methods as a measure of similarity. DEGs were classified into specific biological processes by a Database for Annotation, Visualization, and Integrated Discovery (DAVID) gene ontology (GO) analysis.

### 2.9. Real-Time Quantitative PCR

Total RNA was extracted from hPVCs, lung tissues and alveolar organoids (AOs) using an RNeasy Mini kit (Qiagen, MD, USA). cDNA was synthesized using the TOPscrip^TM^ RT DryMIX (Enzynomics, Daejeon, Korea). PCR amplification was performed using a Step One Plus real-time PCR system (Applied Biosystems, Warrington, UK) with TOPreal^TM^ qPCR 2× PreMIX (Enzynomics, Daejeon, Korea). Relative expression was normalized against GAPDH expression by the ∆∆Ct method. Sequences of primers used in this study are listed in Supplementary Materials Table S1.

### 2.10. Chromatin Precipitation (ChIP)

Chromatin precipitation (ChIP) analysis was performed using Magna ChIP™ G kit (Upstate-Millipore, Billerica, MA, USA) as described previously [16]. The cross-linked chromatins from the indicated cells (1 × 10^7^) were sheared by a Bioruptor Plus sonication device (Diagenode Inc., Denville, NJ, USA) with a standard setting (four 20-s pulses in between 30-s rest intervals on ice) in 500 μL of Nuclear Lysis Buffer. Immunoprecipitation was performed using Protein G magnetic beads, conjugated with 3 μg ChIP grade antibodies against H3Ac (06-599; Millipore, Burlington, MA, USA), H3K4me3 (ab8580; Abcam, Cambridge, UK), H3K27me3 (07-449; Millipore, Burlington, MA, USA) and rabbit immunoglobulin (Ig)G control antibodies (Sigma-Aldrich, Burlington, MA, USA). The enrichment of each histone modification was calculated as the ratio of bound to unbound amplicon fractions and represented as the mean ± SEM from three independent experiments. GraphPad Prism 7.0 software (GraphPad Software, La Jolla, CA, USA) was used to assess statistical significance by a one-way ANOVA followed by Bonferroni post-hoc tests. A *p*-value < 0.05 was considered statistically significant. The sequences of the primers used in the ChIP assay are indicated as follows: JAM2_qChIP_F (AATTCTTTCCAGACGTCTTGACTTC) and JAM2_qChIP_R (AGCTCACTTGTTAAGACCCCCTTA).

### 2.11. Western Blot Analysis

Lung tissues were homogenized with a Polytron homogenizer. Protein extracts were lysed by protein lysis buffer and quantified using the Bicinchonic acid protein assay. Samples (20 μg) were separated by Sodium dodecyl sulphate-polyacrylamide gel electrophoresis (8–15%) gel and then transferred to polyvinylidene fluoride membranes. Nonspecific binding proteins were blocked with 5% skim milk for 1 h at RT. The membranes were incubated with primary antibodies against anti-α-SMA (SC-53015; Santa Cruz Biotechnology, TX, USA) and anti-JAM2 (ab139645; Abcam, Cambridge, UK) overnight at 4 °C. The chemiluminescence signal was scanned using the ChemiDOC^TM^ imaging system (Bio-Rad Laboratories, Hercules, CA, USA).

### 2.12. Generation of Multicellular AOs from hPSCs and HG Treatment

A stepwise direct alveolar epithelial cell (AEC) differentiation and AO generation were performed by a combination of previously reported protocols with minor modifications [17,18]. Briefly, undifferentiated hPSCs were dissociated and then seeded in plates coated with vitronectin at a density of 1 × 10^5^ cells/cm^2^. After an overnight incubation, AEC differentiation was initiated with exposure to sequential induction medium. For the generation of multicellular AOs, the cultures were dissociated on day 14 of AEC differentiation with 0.4 U/mL collagenase B (Roche Diagnostic Inc., Seoul, Korea) for 2 h in a 37 °C incubator, followed by treatment with cell dissociation buffer (Gibco, Waltham, MA, USA) for 10 min in a 37 °C water bath to singularize the cells. The single-cell suspension was then passed through a 70-μm cell strainer (BD Bioscience, CA, USA) and seeded into 96-well round-bottom plates (5 × 10^4^ cells per well) (Corning, NY, USA) containing AEC maturation medium supplemented with 10 μM Rho-associated, coiled-coil containing protein kinase (ROCK) inhibitor (STEMCELL Technologies, Vancouver, BC, Canada). After distribution, 150 μL of 1:15 diluted Matrigel was added into each well to improve adhesion between cells. The plates were centrifuged at 450× *g* for 5 min and incubated overnight to allow aggregation at 37 °C in a humidified atmosphere with 5% CO_2_. After overnight culture, the aggregates were transferred to 6-well low-attachment plates (Corning, NY, USA) containing fresh AEC maturation medium and cultured for 6 days to establish AOs. Then, AOs were treated with 75 mM D-glucose for 6 days.

### 2.13. Statistical Analysis

Values for all measurements are presented as the mean ± standard deviation (SD). Statistical significance was determined using Student’s *t*-test, with *p* < 0.05 considered statistically significant.

## 3. Results

### 3.1. High Glucose Inhibits the Proliferation of hPVCs

**Figure 1:**
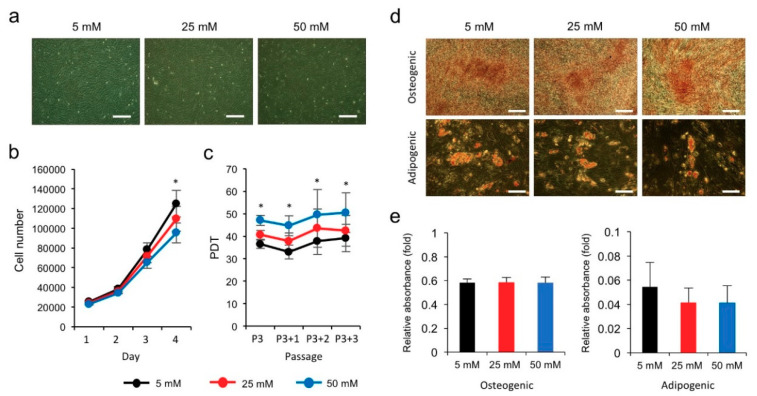
Effects of high glucose on the proliferation and differentiation of human perivascular cells (hPVCs). (**a**) Representative images of hPVCs cultured under normal (5 mM) and hyperglycemic (25 and 50 mM) conditions. Scale bars, 100 μm. (**b**) Growth curve assay of hPVCs cultured under normal glucose (NG) (5 mM) and high glucose (HG) (25 and 50 mM) for 4 days. hPVCs were seeded at a density of 4 × 10^4^ cells per well and counted at 1 to 4 days after culture at each condition. (**c**) PDT was determined from passage 3 (P3) to P6. (**d**) Representative images of Alizarin Red S staining for osteocytes and Oil Red O staining for adipocytes. Scale bars, 100 μm. (**e**) Measurements of Alizarin Red S and Oil Red O contents using spectrophotometry. Bars indicate mean ± SD. * *p* < 0.05.

Since PVCs are known as the primary target of hyperglycemia-induced damage in multiple tissues, we employed hPVCs to identify hyperglycemia-regulated genes using a DNA microarray. Before performing microarrays, we first compared the effects of NG (5 mM) and HG (25 and 50 mM) on the short-term (4 days) and long-term (four passages) proliferative and differentiation capacity of hPVCs. The cells in the HG treatment groups exhibited an elongated and fibroblast-like morphology (Figure 1a). The cell count was significantly lower in HG-treated hPVCs compared to NG-treated cells (Figure 1b). The proliferation rates in both NG- and HG-treated hPVCs were not significantly changed for three passages (from passage 3 to 6). However, the population doubling time (PDT) of hPVCs treated with HG was significantly longer than that of cells treated with NG at all passages analyzed (Figure 1c). We then compared the abilities of NG- and HG-treated hPVCs differentiated into adipocytes and osteocytes. Spectrophotometric quantification revealed no significant differences in both adipogenic and osteogenic inductions (Figure 1d,e). These results suggest that hyperglycemia led to adverse effects on the proliferation of hPVCs, but did not significantly reduce their differentiation potential.

### 3.2. Hyperglycemia Alters Gene Expression Profiles in hPVCs

**Figure 2:**
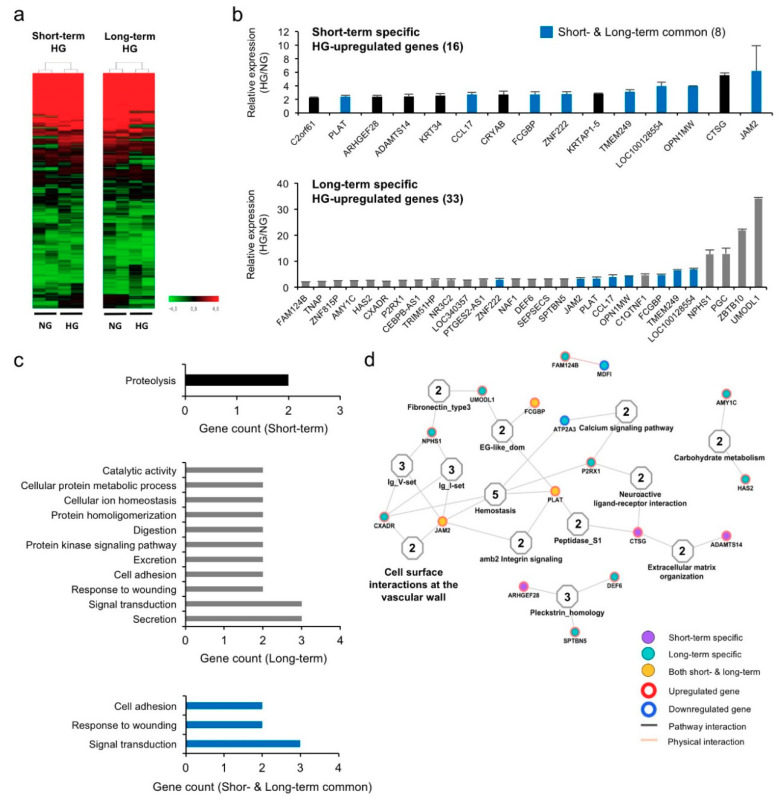
Short-term and long-term effects of HG treatment on gene expression in hPVCs. (**a**) Heatmap of gene expression and unsupervised hierarchical cluster analysis. hPVCs were cultured under NG and HG conditions for 5 days (short-term) and 25 days (long-term) and subjected to a gene expression analysis using a microarray. The color spectrum from green to red indicates low to high expression. (**b**) Graphs for the significantly upregulated genes in hPVCs cultured in HG condition for short- and long-term period, Bars indicate the mean ± SD. (**c**) Short-term and long-term specific gene expressions of hPVCs were analyzed different functions by DAVID gene ontology (GO) analysis. (**d**) Interactions and functional network identified by DAVID GO analysis showed that each of the short- and long-term specific genes was associated pathway and physical interaction of functions.

To understand the hyperglycemia-induced alterations in hPVCs at the molecular level, the expression profiles of two independent hPVC lines treated with NG (5 mM) and HG (50 mM) for 5 days (short-term, ST) and 25 days (long-term, LT) were analyzed using a cDNA microarray with 49,293 human cDNA probes. Unsupervised hierarchical clustering showed that the global expression patterns of PVCs were significantly altered by hyperglycemic conditions (Figure 2a). Among genes with a twofold or greater upregulation relative to the control, 15 and 29 genes were significantly upregulated in ST and LT HG-treated groups, respectively, compared to the corresponding NG-treated groups (Figure 2b; Tables S2 and S3). Among these genes, eight genes (*FCGBP*, *LOC100128554*, *PLAT*, *ZFN222*, *CCL17*, *JAM2*, *OPN1MW*, and *TMEM249*) were found to be commonly upregulated in both groups (blue bars, Figure 2b). To assess the biological relevance of these specific ST and LT upregulated genes, gene ontology (GO) analysis was performed. The dominant functions of the commonly upregulated genes were associated with cell adhesion (*FCGBP* and *JAM2*), wounding (*PLAT* and *CCL17*) and cell surface receptor-linked signal transduction (*PLAT*, *OPN1MW* and *CCL17*) (Figure 2c). The interaction analysis indicated that JAM2 and PLAT are related to other proteins playing important roles in the hemostasis and cell surface interactions at the vascular wall (Figure 2d).

### 3.3. Hyperglycemia-Induced JAM2 Expression Is Transcriptionally and Epigenetically Reversible

**Figure 3:**
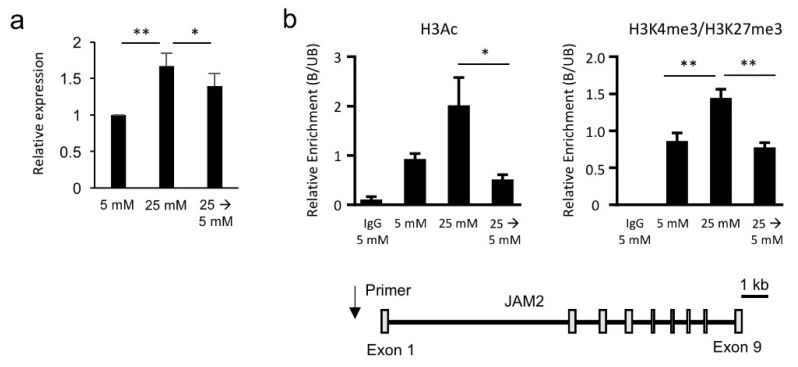
Junctional adhesion molecule 2 (JAM2) is a hyperglycemia-regulated gene in hPVCs. (**a**) qPCR analysis for *JAM2* expression levels in hPVCs cultured in NG (5 mM) and HG (25 mM) for 15 days. hPVC were exposed in HG (25 mM) medium for 5 days followed by NG (5 mM) medium for 10 days. Switch HG to NG medium significantly decreased *JAM2* expression. Bars indicate the mean ± SD. * *p* < 0.05, ** *p* < 0.01. (**b**) qChIP analysis of acetylated lysine 3 of histone 3 (H3Ac) or ratio of tri-methylation at the 4th lysine residue of the histone 3 (H3K4me3) to trimethylated lysine 27 of histone 3 (H3K27me3) in the indicated cells. Data are represented as the ratio to NG (5 mM) exposed cells and are shown as the mean ± SEM (*n* = 3). * *p* < 0.05, ** *p* < 0.01, One-way ANOVA with Bonferroni post-hoc tests. The location of the primer set used in the ChIP assay is marked with a black arrow in the snapshot of the human *JAM2* locus.

To further investigate whether the hyperglycemia-upregulated genes identified from microarray data analysis were conserved in other hPVC lines, we employed four additional hPVC lines for the validation. qPCR analysis showed that seven genes were significantly upregulated (*UMODL1*, *ZFN815P*, *FCGBP*, *NPHS1*, *HAS2*, *PLAT* and *JAM2*) in all hPVC lines treated with HG (25 mM) (Figure 3a and Figure S1). Notably, switching HG (25 mM) medium to NG (5 mM) medium significantly suppressed the transcriptional upregulation of *HAS2*, *PLAT* and *JAM2* (Figure 3a and Figure S1). Based on previous findings showing the potential role of JAM2 in the pathophysiology of DM [19], we next asked if there is an epigenetic mechanism underlying the alteration of *JAM2* expression by HG. We observed increased levels of H3Ac and H3K4me3 enrichment on the *JAM2* locus in HG-treated hPVCs compared to NG-treated cells (Figure 3b). In accordance with transcriptional reversal of *JAM2* by switching HG to NG, H3Ac and H3K4me3 levels on the *JAM2* locus were reduced when HG treatment was removed (Figure 3b). These results demonstrate that hyperglycemia-induced *JAM2* expression is transcriptionally and epigenetically reversible.

### 3.4. Enhanced JAM2 Expression and Histophysiological Alterations in Lung Tissue of Diabetic Mice Were Reduced via Paracrine Action of MSCs

**Figure 4:**
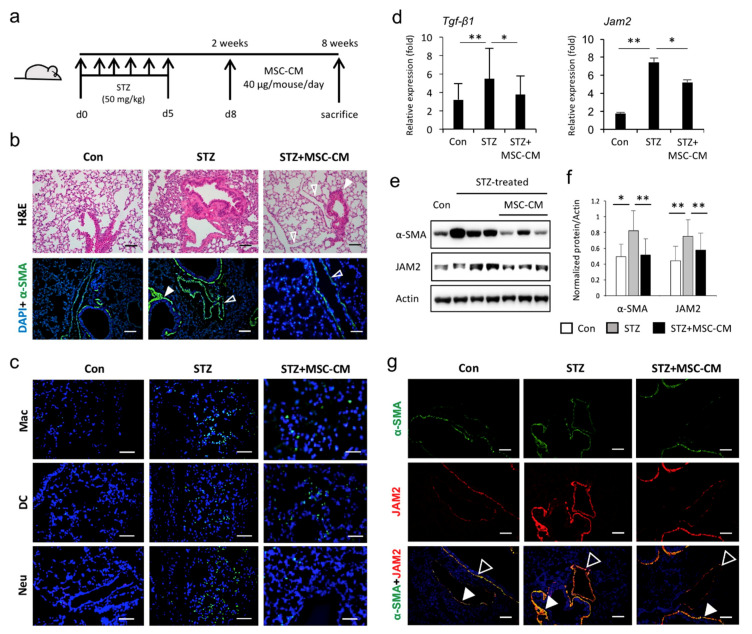
JAM2 is upregulated in streptozotocin (STZ)-induced diabetic mouse lung tissue. (**a**) A schematic of hyperglycemia induction using STZ and the conditioned medium from human mesenchymal stem cells (MSC-CM) treatment protocol. (**b**) Hematoxylin and eosin (H&E) and immunofluorescence staining for alpha smooth muscle actin (α-SMA) (green) in lung sections from the Con, STZ, and STZ + MSC-CM groups. Nuclei were counterstained with DAPI (blue). Closed arrowheads indicate bronchioles while open arrowheads indicate vessels. Scale bars, 50 μm. (**c**) Immunostaining of leukocytes including macrophages (Mac), dendritic cells (DC) and neutrophils (Neu) in the lung sections from each group. Scale bars, 50 μm. (**d**) qPCR analysis for *Tgf-β1* and *Jam2* expression levels in lung tissue from each group. Bars indicate the mean ± SD. * *p* < 0.05, ** *p* < 0.01. (**e**) Western blot analysis of the expression of α-SMA and JAM2 proteins in whole lung homogenates of mice from the indicated group. (**f**) Quantitative analysis shows the relative percentage of α-SMA and JAM2 protein expression levels. (**g**) Immunofluorescence staining for α-SMA (green) and JAM2 (red) in lung sections from each group. Closed arrowheads indicate bronchioles while open arrowheads indicate vessels. Scale bars, 50 μm. Bars indicate the mean ± SD. * *p* < 0.05, ** *p* < 0.01.

To further investigate how the diabetic condition and multipotent stem cells regulate *JAM2* expression in vivo, we utilized lung tissues from an STZ-induced diabetic mice model with or without the treatment of MSC-CM (Figure 4a). As previously reported [20], hyperglycemia resulted in alveolar wall thickening and a reduction in alveolar air space (Figure 4b). Furthermore, immunofluorescence staining showed a brighter fluorescence intensity for α-SMA in pulmonary vessel areas of diabetic mice compared to the control mice and a massive influx of leukocytes (macrophages, neutrophils and dendritic cells) into the lung tissues of diabetic mice (Figure 4b,c). We also found that these STZ-induced histological and physiological alterations in lung tissues were reversed by administration of MSC-CM (Figure 4b,c). In addition, elevated levels of inflammatory *Tgf-β1* cytokine in lungs of STZ-induced diabetic mice were significantly reduced by MSC-CM treatment (Figure 4d). Importantly, hyperglycemic conditions stimulate enhanced expression of *Jam2* at the mRNA and protein levels, which are effectively reduced by MSC-CM treatment (Figure 4d–f). The immunostaining of lung tissues demonstrated that JAM2 and α-SMA were co-localized to the smooth muscle layer of bronchioles and the medial vessel layer and strongly detected in these sites of the STZ-treated group, which were effectively reduced by MSC-CM treatment (Figure 4g). The recent COVID-19 pandemic prompted us to examine the expression level of angiotensin-converting enzyme 2 (*Ace2*), the cellular receptor for severe acute respiratory syndrome coronavirus clade 2 (SARS-CoV-2), and the cofactor transmembrane protease serine 2 (*Tmprss2*) in the lung tissues of STZ-induced diabetic mice. Interestingly, the transcription level of *Tmprss2* was significantly increased in the lung tissue of STZ-induced diabetic mice compared to the control (Figure S2). These results suggest that *JAM2* may play an important role in STZ-induced lung alterations and could be a potential indicator for predicting the therapeutic effects of stem cells as well as the development of fibrosis in diabetic lung complications.

### 3.5. Hyperglycemia Induces JAM2 Upregulation in hPSC-Derived AOs

**Figure 5:**
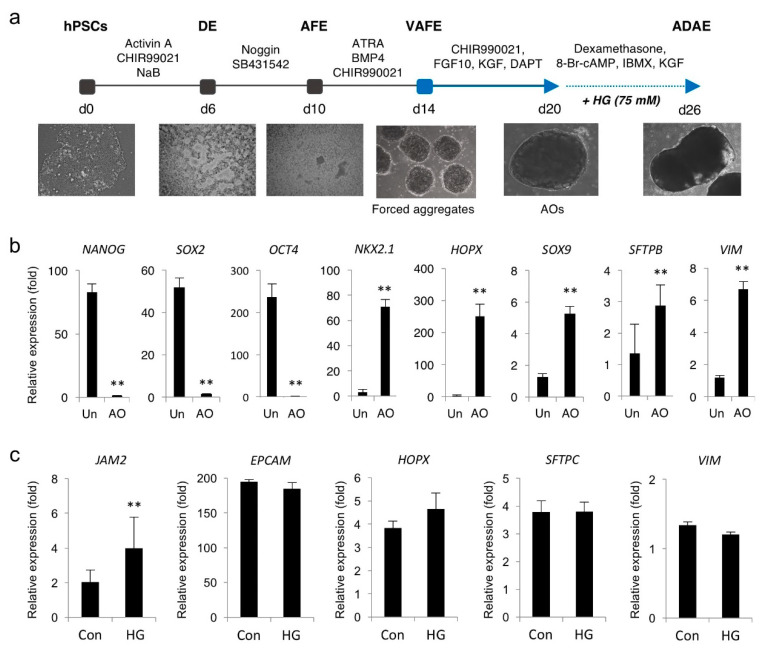
High glucose upregulated JAM2 expression in human AOs. (**a**) Schematic overview of the generation of AOs from hPSCs. (**b**) qPCR of the indicated pluripotency and AEC markers in AOs. Data are shown as fold-change relative to undifferentiated hPSCs (Un). Data are presented as mean ± SD. ** *p* < 0.01. (**c**) qPCR analysis of JAM2 and AEC markers in the control (17.5 mM) and HG (75 mM)-treated AOs. Data are presented as mean ± SD. ** *p* < 0.01. DE, definitive endoderm; AFE, anterior foregut endoderm; VAFE, ventral anterior foregut endoderm; ADAE, alveolar and distal airway epithelium.

Next, we investigated the hyperglycemic regulation of JAM2 using an in vitro human AO model generated from hPSCs. Previously, we and others reported efficient protocols for the production of functional AECs from hPSCs and the formation of cellular aggregates from a single cell suspension of hPSCs by forced aggregation [17,18] (Figure 5a). By taking advantage of these protocols, multicellular AOs containing alveolar progenitors (*NKX2.1*), AECs (*HOPX*, *SOX9* and *SFTPB*) and stromal cells (*VIM*) were generated and tested for *JAM2* expression under hyperglycemic conditions (Figure 5b). We found that AOs treated with HG (75 mM) for 6 days exhibited the significant upregulation of *JAM2* without affecting expression levels of AECs and stromal cell markers (Figure 5c). These findings indicate that hyperglycemic conditions induced *JAM2* upregulation in alveolar tissue derived from hPSCs, which may serve as in vitro models for understanding the pathological development of diabetic lung complications.

## 4. Discussion

Chronic hyperglycemic conditions result in histological and physiological alterations in the lung tissues, which have a positive correlation with an increased risk of various pulmonary diseases including asthma, COPD and PF [9,10]. Thus, identifying the genes associated with STZ-induced lung alterations is of major importance for understanding the pathophysiology underlying lung dysfunction and facilitating the development of novel therapeutic strategies. We have shown, in the current study, that JAM2 was elevated at the transcriptional and translational level in the lung tissue of type 1 diabetic mice. Although the underlying mechanisms to explain the potential role of JAM2 and its regulation in diabetic lung tissue have not been clarified in the present study, previous findings suggest that JAM2 might be involved in regulating the inflammatory response and the development of lung fibrosis under hyperglycemic conditions. It is well known that JAMs participate in the regulation of endothelial junctions, leukocyte–vascular wall cell interactions, and control vascular permeability [21,22]. In type 1 diabetic mice, JAM2 and JAM3 have been reported to polarize leukocyte trans-endothelial migration and subsequently destruct insulin-producing beta cells in the pancreas [23]. Thus, the blocking of JAMs efficiently attenuated autoimmune diseases such as pancreatitis and rheumatoid arthritis by inhibiting interactions between immune cells and vessel components. Moreover, the upregulation of JAM2 and JAM3 by endothelial cells and de novo synthesis of JAM3 by hepatic stellate cells trigger autoimmune-mediated liver fibrosis in mice [24]. Our findings, together with those of others, suggest that JAM2 may play an important role in STZ-induced lung alterations and could be a therapeutic target for diabetic lung complications.

Fibrosis is the last stage of chronic inflammatory responses and is characterized by the excessive deposition of the extracellular matrix, leading to tissue dysfunction in the affected organs [25]. Accumulating evidence suggests that MSCs have great potential in the treatment of fibrosis due to their abilities to act on proinflammatory and profibrotic factors such as oxidative stress, the TGF-β signaling pathway, and hypoxia [26,27,28]. Consistent with previous studies, we found that diabetic mice appeared to have a thickening alveolar wall, increased expression of SMA, and a massive infiltration of leukocytes in the lung tissue, exhibiting an obvious fibrosis. Thus, although the therapeutic efficacy of MSCs was somewhat predictable, this is the first report to show the amelioration of STZ-induced lung alterations by the administration of human MSC-CM. Notably, MSC-CM suppressed hyperglycemia-enhanced JAM2 expression in the lungs, suggesting that JAM2 could be a useful indicator for predicting the in vivo therapeutic outcomes of MSCs isolated from different donors and different tissue sites for diabetic lung fibrosis.

Although MSCs have been intensively explored as promising therapeutic agents for the treatment of various diseases, the low survival rate of transplanted MSCs in the recipient tissues and lower receptivity of the damaged tissues to the transplanted MSCs are yet to be overcome. Recent studies demonstrated that the benefits of MSC therapy could be due to their secretions, which play an important role in the regeneration of damaged tissues and may present considerable advantages over cell-based applications for manufacturing, handling and storage [29]. The possible therapeutic effect of MSCs in diabetic complications has also been suggested by the paracrine action of MSC-secreted factors (e.g., the secretome) as well as their capacity to generate insulin-producing cells [30] Moreover, the secretome is affected by the preconditioning of MSCs during isolation and maintenance [31,32] One paracrine mechanism of MSCs involves the secretion of extracellular vesicles (EVs), which are able to transfer various materials to recipient cells [33,34,35]. The present study showed that enhanced JAM2 expression and histophysiological alterations in lung tissue of diabetic mice were reduced via the paracrine action of MSCs. Thus, identifying specific soluble factors from MSC-CM or -EVs and preconditioning to improve their paracrine activity will facilitate the development of novel therapeutic strategies for diabetic lung complications.

hPSCs have been proven to be useful resources for modeling various diseases due to their unique ability to differentiate into all cell types in the body [36]. However, the conventional two-dimensional (2D) cell culture system is limited to recapitulating the structure and functions of in vivo tissues. Thus, the need for a more accurate model system has moved the field towards developing 3D organoids from hPSCs, which are able to overcome the limited accessibility to primary normal human and diabetic lung tissues and provide a more faithful representation of native lung tissue [37]. In the lungs, several types of organoids that represent different respiratory compartments such as the proximal and distal airways have been successfully generated from hPSCs and already proven to be a great platform for the modeling of human lung development and pulmonary fibrosis [38,39,40,41]. However, no studies have been conducted to investigate the effects of hyperglycemic conditions on gene expression using hPSC-derived AO. In the present study, hyperglycemic conditions induced JAM2 upregulation in hPSC-derived AOs, indicating that hyperglycemia-induced JAM2 expression might be conserved between murine animals and humans. Although AOs used in this study contain multiple alveolar cell types, the AOs remain incomplete as they lack vasculature and tissue resident immune cells, which create the critical microenvironment relevant for developmental and pathological processes. Thus, the development of AOs closer to the native tissue architecture and functions by the incorporation of key components is essentially required to provide a more robust in vitro platform for studying the cellular and molecular mechanisms of diabetic lung complications.

## 5. Conclusions

Taken together, the current study suggested that JAM2 may play an important role in STZ-induced lung alterations and could be an indicator to predict the therapeutic effects of multipotent stem cells. Further investigations will be necessary to fully reveal the underlying molecular mechanisms, but JAM2 is undoubtedly a potential target worth further research in treating diabetic lung complications.

## Supplementary Materials

Supplementary Materials can be found at https://www.mdpi.com/2227-9059/8/9/346/s1.
