# Supplementary material for: Regulation of JAM2 Expression in the Lungs of Streptozotocin-Induced Diabetic Mice and Human Pluripotent Stem Cell-Derived Alveolar Organoids
Source: Biomedicines. 2020 Sep 11;8(9):346. doi: 10.3390/biomedicines8090346 (PMC7555027; doi:10.3390/biomedicines8090346)
Supplement: Supplementary file 1 [file biomedicines-08-00346-s001.pdf]

Supplementary Information

Regulation of JAM2 expression in the lungs of streptozotocin-induced diabetic mice and human pluripotent stem cell-derived alveolar organoids

Roya Rasaei, Eunbi Kim, Ji-Young Kim, Sunghun Na, Jung-Hyun Kim, Jinbeom Heo, Dong-Myung Shin, Sun Shim Choi, Seok-Ho Hong

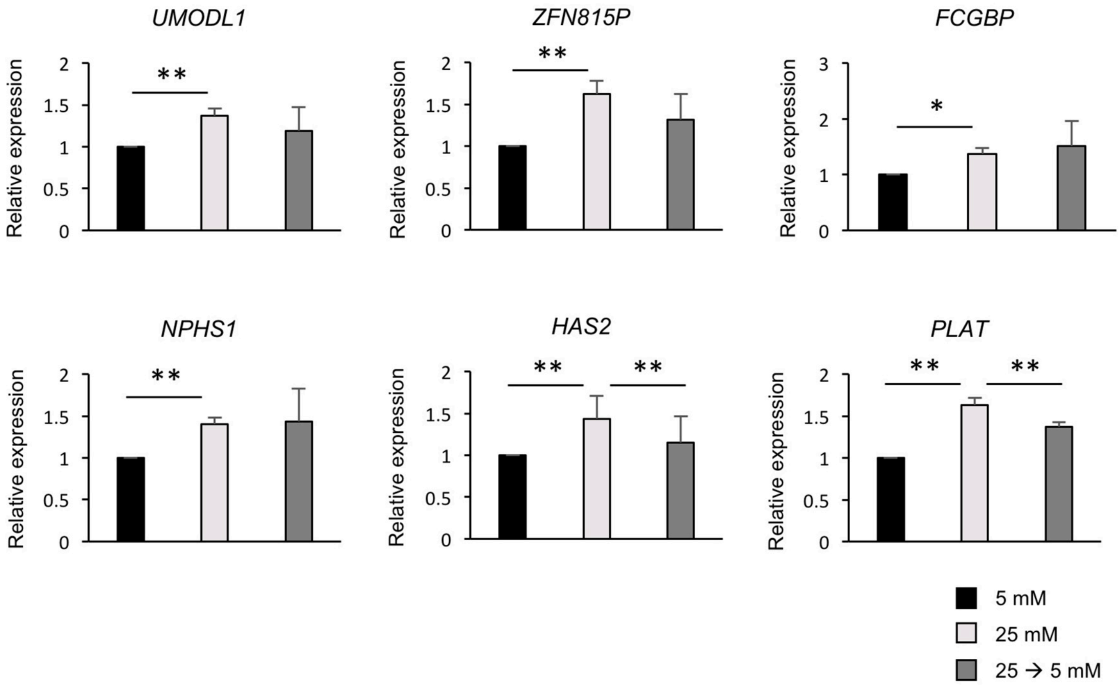

Figure S1. Validation of HG-regulated genes in hPVCs. qPCR was used for the verification of upregulated genes in HG-treated hPVCs using four independent hPVC lines. *JAM2*, *HAS2* and *PLAT* were upregulated in hPVC lines by HG exposure and were partially reversible when HG treatment was removed. Bars indicate the mean ± SD. *p<0.05, **p<0.01.

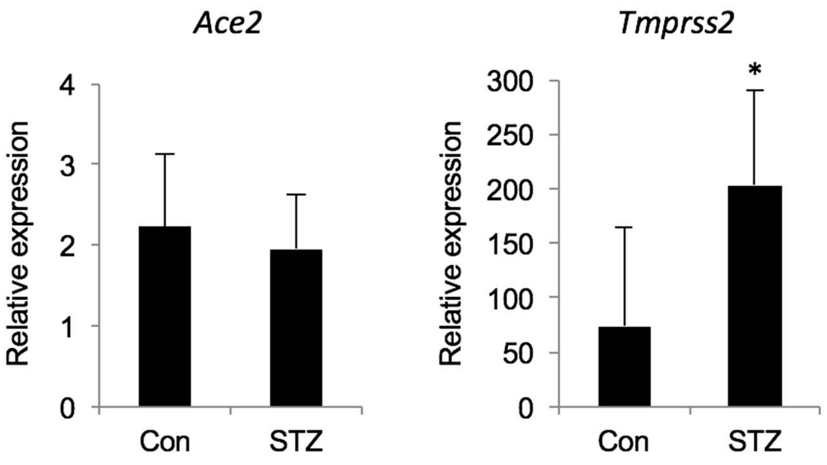

Figure S2. Expression of *Ace2* and *Tmprss2* in the lungs of STZ-induced diabetic mice. qPCR analysis of *Ace2* and *Tmprss2* expression in the lung of STZ-induced diabetic mice. Bars indicate the mean ± SD. *p<0.05.

Table S1. Human and mouse primer sequences used for qPCR

Genes		Sequence 5' to 3'	Product size (bp)
hJAM2	F	GTGGCCTTGGTGTATGCTAT	100
	R	TCACTCATTGTCGTGGCTTT	
hSFTPB	F	GCC ATA CCA CAG GCA ATG CT	80
	R	TGC TGC TCC ACA AAT TGC TT	
hSFTPC	F	CCT TCT TAT CGT GGT GGT GGT	96
	R	TCT CCG TGT GTT TCT GGC TCA T	
hSOX9	F	GTACCCGCACTTGCACAAC	107
	R	GTGGTCCTTCTTGTGCTGC	
hHOPX	F	GCC TTT CCG AGG AGG AGA C	97
	R	TCT GTG ACG GAT CTG CAC TC	
hEPCAM	F	AGAACCTACTGGATCATCATTGAAC TAA	101
	R	CGCGTTGTGATCTCCTTCTG	
hNKX2.1	F	AGC ACA CGA CTC CGT TCT CA	75
	R	CCT CCA TGC CCA CTT TCT TG	
hVIMENTIN	F	CCA GGC AAA GCA GGA GTC	212
	R	CGA AGG TGA CGA GCC ATT	
hα-SMA	F	GAC GAA GCA CAG AGC AAA AG	70
	R	AGT TGG TGA TGA TGC CAT GT	
hSOX2	F	GCACATGAAGGAGCACCCGGATTA	86
	R	CGGGCAGCGTGTACTTATCCTTCTT	
hNANOG	F	CAAAGGCAAACAACCCACTT	158
	R	TCTGCTGGAGGCTGAGGTAT	
hOCT4	F	TCGAGAACCGAGTGAGAGG	125
	R	GAACCACACTCGGACCACA	
hUMODL1	F	CCACACCAGCCCTCAGAAC	100
	R	CGGTCGGGTCTCTCTGAGAA	
hZNF222	F	CCACACGGGAGAGAGATCTT	100
	R	CTTTCTTTGGCAGTGGAGTT	
hFCGBP	F	ACCGTGTAGTTGCCGAAGTC	100
	R	TTTGGAGTCAACGTCACCAT	
hNPHS1	F	TCCACTGGCCTGAAGACACA	100
	R	AGCTCGAAGGGCAGAGAATC	
hPLAT	F	AGATGGGAAGACATGAATGC	104
	R	GTCAGGAGGTTGGGCTTTAG	

hHAS2	F	GAAAAGGGTCCCGGTGAGAC	259
	R	CCCCAACACCTCCAACCATG	
hGAPDH	F	GGC ATG GAC TGT GGT CAT GA	87
	R	TGC ACC ACC AAC TGC TTA GC	
mGapdh	F	GTTGTCTCCTGCGACTTCA	184
	R	GGTGGTCCAGGGTTTCTTA	
mJam2	F	ACGAAGCTTTCAATATACGAATCAAAA	334
	R	CATGTTGAATTGCAGAATTC	
mActa2 (α-Sma)	F	CCCCTGAAGAGCATCGGACA	105
	R	: TGGCGGGGACATTGAAGGT	

Table S2. List of genes upregulated by short-term HG treatment in PVCs

Genes	Name	GeneBank	Fold change (HG/LG)
JAM2	Junctional adhesion molecule 2	NM_021219	6.220
CTSG	Cathepsin G	NM_001911	5.559
OPN1MW	Opsin 1 (cone pigments), medium-wave-sensitive	NM_000513	3.974
LOC100128554	Uncharacterized LOC100128554	NR_015398	3.936
TMEM249	Transmembrane protein 249	NM_001280561	3.099
KRTAP1-5	Keratin associated protein 1-5	NM_031957	2.822
ZNF222	Zinc finger protein 222	NM_013360	2.747
FCGBP	Fc fragment of IgG binding protein	NM_003890	2.741
CRYAB	Crystallin, alpha B	NM_001885	2.711
CCL17	Chemokine (C-C motif) ligand 17	NM_002987	2.699
KRT34	Keratin 34, type I	NM_021013	2.564
ADAMTS14	ADAM metallopeptidase with thrombospondin type 1 motif, 14	NM_139155	2.437
ARHGEF28	Rho guanine nucleotide exchange factor (GEF) 28	NM_001177693	2.395
PLAT	Plasminogen activator, tissue	NM_000930	2.372
C2orf61	Chromosome 2 open reading frame 61	NM_173649	2.260

Table S3. List of genes upregulated by long-term HG treatment in PVCs

Genes	Name	GeneBank	Fold change (HG/LG)
UMODL1	Uromodulin-like 1	NM_173568	34.178
ZBTB10	Zinc finger and BTB domain containing 10	NM_001105539	21.807
PGC	Progastricsin (pepsinogen C)	NM_002630	12.807
NPHS1	Nephrosis 1, congenital, Finnish type (nephrin)	NM_004646	12.671
C1QTNF1	C1q and tumor necrosis factor related protein 1	NM_198594	6.760
LOC100128554	Uncharacterized LOC100128554	NR_015398	6.406
OPN1MW	Opsin 1 (cone pigments), medium-wave-sensitive	NM_000513	4.658
FCGBP	Fc fragment of IgG binding protein	NM_003890	4.640
TRIM51HP	Tripartite motif-containing 51H, pseudogene	ENST00000530412	4.196
TMEM249	Transmembrane protein 249	NM_001280561	3.922
PLAT	Plasminogen activator, tissue	NM_000930	3.367
DEF6	Differentially expressed in FDCP 6 homolog (mouse)	NM_022047	3.174
PTGES2-AS1	PTGES2 antisense RNA 1 (head to head)	NR_024425	3.154
JAM2	Junctional adhesion molecule 2	NM_021219	3.064
CCL17	Chemokine (C-C motif) ligand 17	NM_002987	3.044
ZNF222	Zinc finger protein 222	NM_013360	3.014
ZNF815P	Zinc finger protein 815, pseudogene	NR_023382	2.941
TNAP	TRAFs and NIK-associated protein	AF463496	2.797
SPTBN5	Spectrin, beta, non-erythrocytic 5	NM_016642	2.710
SEPSECS	Sep (O-phosphoserine) tRNA:Sec (selenocysteine) tRNA synthase	NM_016955	2.665
P2RX1	Purinergic receptor P2X, ligand gated ion channel, 1	NM_002558	2.650
CXADR	Coxsackie virus and adenovirus receptor	NM_001338	2.633
CEBPB-AS1	CEBPB antisense RNA 1	NR_125739	2.622
AMY1C	Amylase, alpha 1C (salivary)	NM_001008219	2.397
NR3C2	Nuclear receptor subfamily 3, group C, member 2	NM_000901	2.395
NAF1	Nuclear assembly factor 1 ribonucleoprotein	NM_138386	2.362
FAM124B	Family with sequence similarity 124B	ENST00000243806	2.344
HAS2	Hyaluronan synthase 2	NM_005328	2.149
LOC340357	Uncharacterized LOC340357	NR_015383	2.057
